# The Origin of Minus-end Directionality and Mechanochemistry of Ncd Motors

**DOI:** 10.1371/journal.pcbi.1002783

**Published:** 2012-11-15

**Authors:** Biman Jana, Changbong Hyeon, José N. Onuchic

**Affiliations:** 1 Center for Theoretical Biological Physics, Rice University, Houston, Texas, United States of America; 2 School of Computational Sciences, Korea Institute for Advanced Study, Seoul, Korea; University of Illinois, United States of America

## Abstract

Adaptation of molecular structure to the ligand chemistry and interaction with the cytoskeletal filament are key to understanding the mechanochemistry of molecular motors. Despite the striking structural similarity with kinesin-1, which moves towards plus-end, Ncd motors exhibit minus-end directionality on microtubules (MTs). Here, by employing a structure-based model of protein folding, we show that a simple repositioning of the neck-helix makes the dynamics of Ncd non-processive and minus-end directed as opposed to kinesin-1. Our computational model shows that Ncd in solution can have both symmetric and asymmetric conformations with disparate ADP binding affinity, also revealing that there is a strong correlation between distortion of motor head and decrease in ADP binding affinity in the asymmetric state. The nucleotide (NT) free-ADP (φ-ADP) state bound to MTs favors the symmetric conformation whose coiled-coil stalk points to the plus-end. Upon ATP binding, an enhanced flexibility near the head-neck junction region, which we have identified as the important structural element for directional motility, leads to reorienting the coiled-coil stalk towards the minus-end by stabilizing the asymmetric conformation. The minus-end directionality of the Ncd motor is a remarkable example that demonstrates how motor proteins in the kinesin superfamily diversify their functions by simply rearranging the structural elements peripheral to the catalytic motor head domain.

## Author Summary

Proteins belonging to the kinesin superfamily are responsible for vesicle or organelle transport, spindle morphogenesis, and chromosome sorting during cell division. Interestingly, while most proteins in kinesin superfamily that share the common catalytic motor head domain have plus-end directionality along microtubules, kinesin-14 (Ncd) exhibits minus-end directionality. Despite the several circumstantial evidences on the determining factors for the motor directionality in the last decade, a comprehensive understanding of the mechanism governing the Ncd minus-end directionality is still missing. Our studies provide a clear explanation for this minus-end directionality and the associated mechanochemical cycle. Here, we modeled an Ncd motor by employing structural details available in the literature to simulate its conformational dynamics. Simulations using our structure-based model of Ncd assert that the dynamics due to a simple rearrangement of structural elements, peripheral to the catalytic motor domain, is the key player in determining both the directionality and mechanochemistry unique to Ncd motors. Although Ncd has a different directionality, it uses a similar strategy to kinesin-1 of structural adaptation of the catalytic motor domain. Therefore using the same physical principle of protein folding and very similar structural elements, motors in the kinesin superfamily are able to achieve a variety of biological function.

## Introduction

Motor proteins in the kinesin superfamily play critical roles in a number of cellular processes such as vesicle and organelle transport, microtubule depolarization, chromosome and spindle organization during cell division [1], [2], [3]. They convert chemical energy associated with ATP hydrolysis into mechanical work to undertake their specific tasks in the cell. While sharing a very similar structure, especially in the catalytic motor head (MH) domain, proteins in the kinesin superfamily exhibit stark variations in their biological function [4], [5]. Kinesin-1 moves from the minus-end of MTs to the plus-end [6], [7]; kinesin-5 (or Eg5) assembles antiparallel MTs to organize bipolar spindles [8], [9]; kinesin-13, responsible for the depolymerization of MTs, diffuses bidirectionally along the MT [10]; and kinesin-14 (Ncd) is minus-end-directed [11]. Among many outstanding questions related to motor dynamics [12], it is of particular interest to ask how the directionality of the molecular movement is determined for a given structure of a motor.

Among the motor proteins in the kinesin family, one of the best studied is the kinesin-1 that walks hand-over-hand towards the plus-end of MTs [13], [14], [15]. In kinesin-1, NT-dependent affinity of the MH to MTs and order-disorder transition of the neck-linker, which connects the MH with C-terminal neck-helix, were suggested as the key structural elements responsible for the motor directionality and head-to-head coordination [16], [17], [18], [19], [20], [21], [22], [23], [24]. For a given binding interface between the MH and MTs, ATP-binding induced disorder-to-order transition of the neck-linker rectifies the diffusive dynamics of a tethered head towards the plus-end. In contrast to the kinesin-1, Ncd motors retain its MH next to the N-terminal neck-helix. Cryo-electron microscope studies and a single molecule assay suggest that Ncd generates force from the plus to minus-end using only one head by keeping the remaining head away from the MTs during the entire motor cycle [25], [26], [27]; thus suggesting that the dynamics of Ncd is non-processive [11], [26]. Although the reconstructed images of the kinesin-1 and Ncd on MTs provide partial glimpses of their functional mechanism [25], [26], [27], a further study is needed to address the structural and dynamical origin of opposite directionalities for the two motors.

Several findings from previous experiments provide valuable insights into Ncd dynamics. Stopped-flow experiment by Foster *et al.* suggested an apparent asymmetry between the two MHs in Ncd dimers [28], showing biphasic ADP release kinetics with disparate time scales of 7 s^−1^ and 0.005 s^−1^. An asymmetric Ncd conformer crystallized by mutating a residue (N600K) in the MT binding motif showed a structure, with a considerably low ADP affinity, distorted from the wild type [29], suggesting that such an asymmetric conformation can be accessible for the wild type Ncd. As a plausible link between the biphasic ADP release kinetics and the presence of accessible asymmetric states, ^13^P-NMR spectroscopy showed that multiple Ncd dimer conformations, including asymmetric conformers, are accessible in solution [30]. Furthermore, according to mutation studies, the directionality of motor hinges on the structural elements outside of the MH domain [31]: a chimeric kinesin-1 with Ncd's neck is minus-end-directed, whereas an Ncd with kinesin-1's neck-linker exhibited the plus-end directionality although the motility was impaired in both cases 32,33, which highlights the role of the neck domain for the motor directionality.

Despite a number of studies on Ncd motors, still missing is a more integrated understanding that explicitly relates the scattered experimental findings mentioned above with the mechanochemical cycle of Ncd motors and their minus-end directionality in structural terms. A series of efforts have recently been made to decipher a number of crucially interesting issues involving molecular motors by performing large-scale molecular simulations [19], [20], [24], [34], [35], [36], [37], [38], [39], [40], [41]. Among them, our previous studies that employed the coarse-grained structure-based model of kinesin-1, which retains its native-like configuration in the absence of external perturbation, could show that the conformational adaptation in response to the chemical and mechanical stresses renders the movement of kinesin-1 plus-end directed and highly processive [19], [20], [23]. Here, our model of the Ncd motor, adopting the computational strategy based on the theory of protein folding as the previous models [19], [20], [23], [42], [43], clarifies how the repositioning of neck-helix to the N-terminal and the deletion of neck-linker from kinesin-1 drastically alter the intramolecular structural adaptation, giving rise to the minus-end directionality and associated mechanochemistry unique to the Ncd motors. Finally, our study show explicitly how the basic physical principle of protein folding [19], [20], [23], [44], [45], [46] play roles for motor proteins in the kinesin superfamily to achieve a wealth of different behaviors needed for their biological functions.

## Results

### Structural comparison between kinesin-1 and Ncd

**Figure 1:**
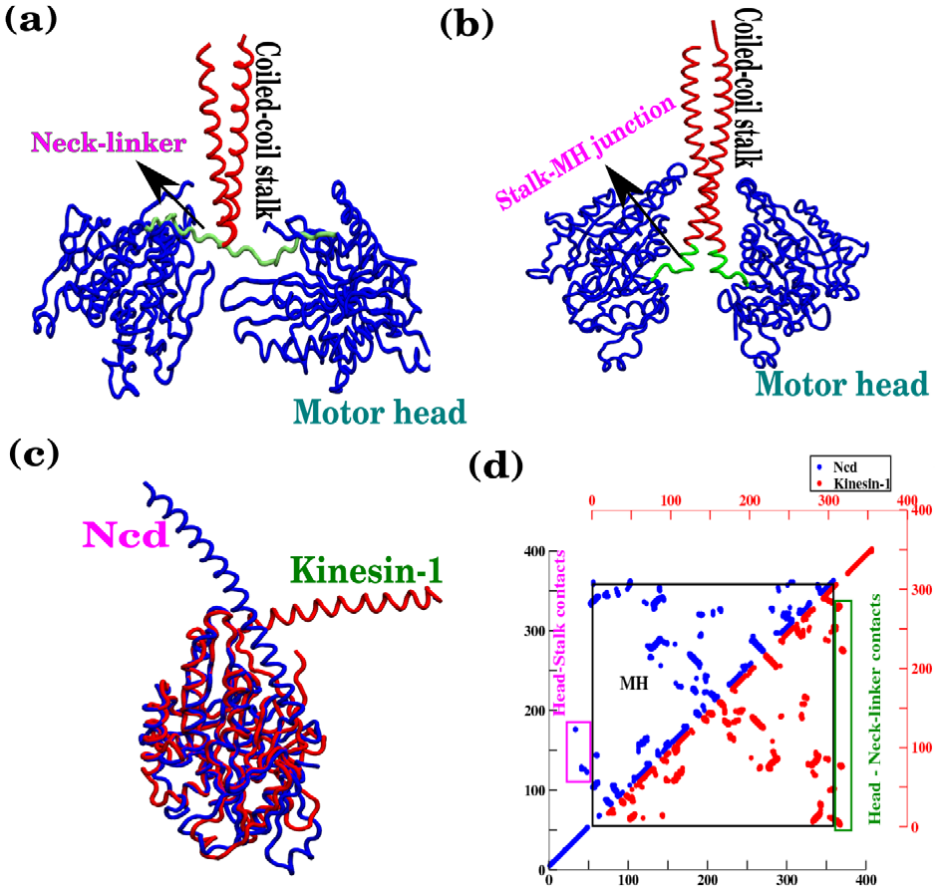
Structural comparison of kinesin-1 and Ncd. (a) Dimer structures of kinesin-1 (PDB ID 3KIN) in ADP-ADP state in the absence of MT. (b) Dimer structure of the symmetric Ncd dimer (PDB ID 1CZ7). Note that the Ncd motor lacks the neck-linker region. (c) Superposition of the MHs of kinesin-1 (red) and Ncd (blue). Between the MHs of the two motors Cα-rmsd is 1.06 Å, and there is 41% sequence identify. (d) Native contact maps of the two motors, upper left for Ncd and lower right for kinesin-1. Note that the patterns of the contact map between the two MH domains enclosed in black box are very similar, and that the differences are in structural motifs peripheral to MH.

Native contact maps and superimposed structures between the kinesin-1 (Fig. 1a) and symmetric conformer of Ncd (Fig. 1b) reveal notable similarity of the two molecules. The MH domains of two motors are very similar in both structure (Fig. 1c) and contact map (Fig. 1d). The main difference between the Ncd and kinesin lies in the organization of structural motifs peripheral to the MH domain. While kinesin-1 has a C-terminal neck-helix followed by neck-linker and MH, Ncd has a N-terminal neck-helix that is linked to the MH via a short stretch of amino acids called neck-junction, which introduces slight variations in the contact maps. It is of particular note that the contacts between the neck-helix (or coiled-coil stalk) and the MH are unique to Ncd (enclosed by the magenta box in Fig. 1d). Instead of these head-stalk contacts, kinesin-1 has multiple contacts between the MH and neck-linker (enclosed by the green box in Fig. 1d). We show that this seemingly minor difference in contact map leads to stark differences in terms of motor functions between Ncd and kinesin-1.

### Transitions between symmetric and asymmetric conformations of Ncd (ADP-ADP state) in solution

**Figure 2:**
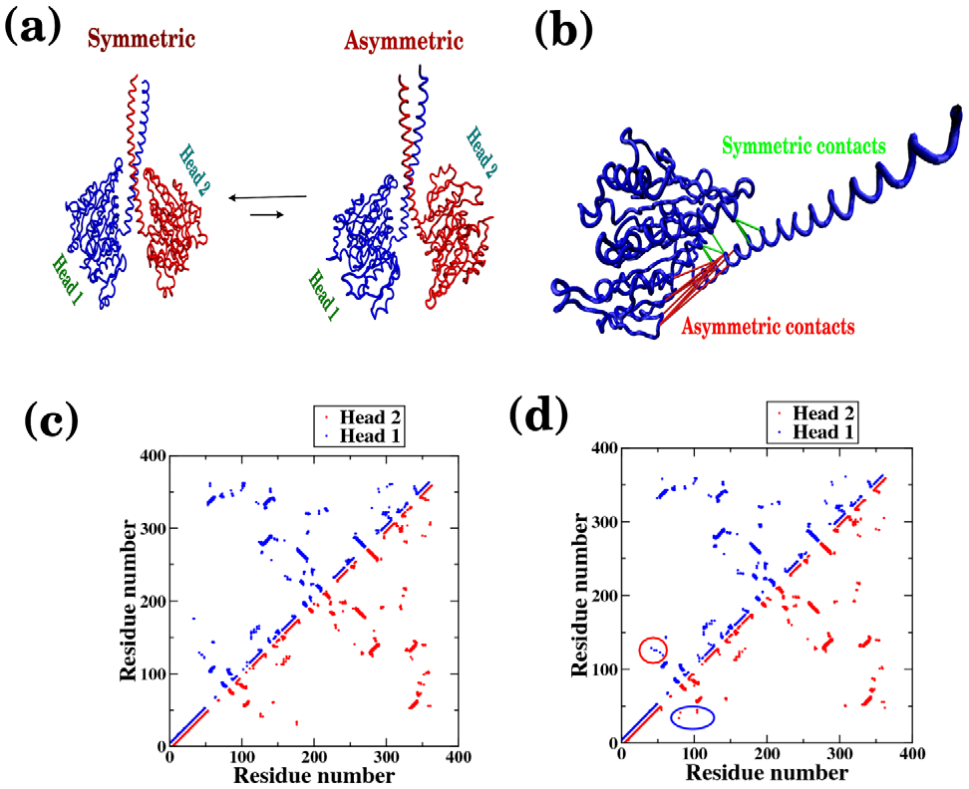
Conformational transition between symmetric and asymmetric conformers of Ncd dimer in the absence of MTs. (a) Ncd motor in the symmetric and asymmetric states. (b) Head-stalk contacts present in the symmetric (green) and asymmetric state (red) shown for a monomer. These two sets of contacts cannot be satisfied simultaneously. Residue contact maps for (c) symmetric and (d) asymmetric conformations. The difference in the head-stalk contacts in the asymmetric conformer is highlighted in the contact map by using circles.

**Figure 3:**
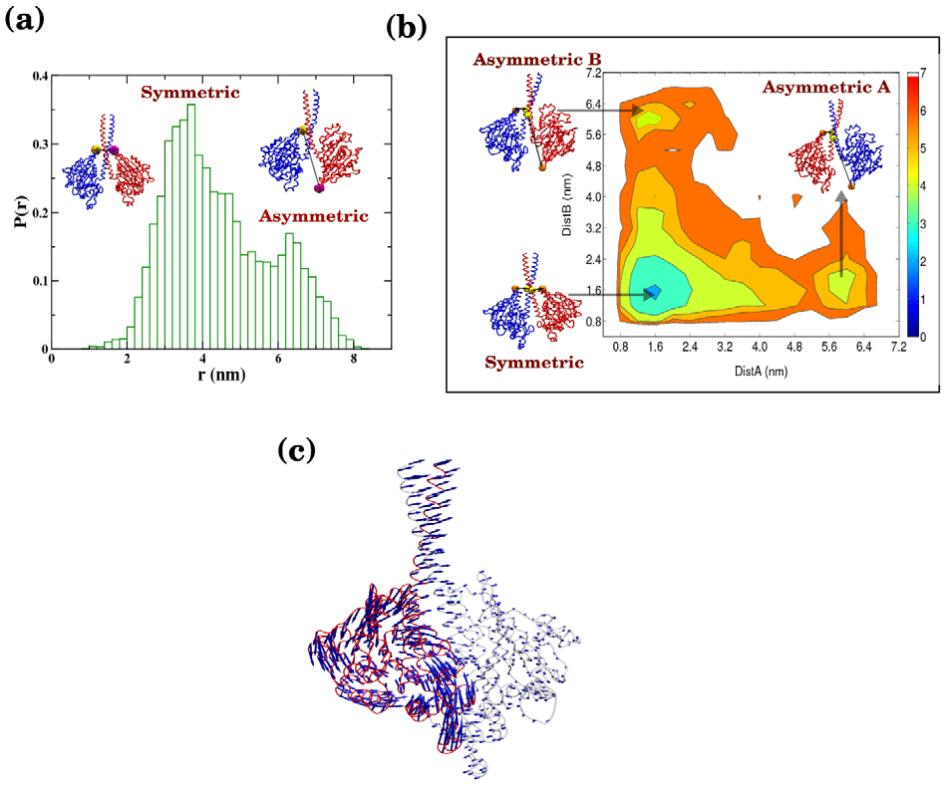
Dynamics between symmetric and asymmetric states of Ncd in solution. (a) Distribution of the inter-residue distance between E567 of the two monomers calculated from simulation show two peaks corresponding to the symmetric and asymmetric states with representative Ncd structures for each peak position. (b) Two dimensional free energy diagram color-coded in *k*_B_ *T* unit. Energetically possible transitions are between symmetric and one of the two asymmetric states (A or B). (c) Low frequency modes from PCA lend support to the distortion dynamics that drives the Ncd dimer from its symmetric to its asymmetric state. Residue-wise vector represents one of the two low frequency modes.

In the asymmetric conformation of Ncd (Fig. 2a), one of the two MHs has a structure rotated around the neck-junction and thus “distorted” from its symmetric counterpart that leads to a different contact map (Fig. 2c & d). As we have discussed earlier, wild type Ncd can exists in either the symmetric or asymmetric form in solution. In our structure-based potential we combine the information of the native contacts from both symmetric and asymmetric states. As clearly depicted in the Ncd structure of Fig. 2b, it is impossible for the stalk (residue indices around 50) to form contacts concurrently with MH in the symmetric state (residue indices around 125) and in the asymmetric state (residue indices 80–100). Thus, the head-stalk contacts in the symmetric and asymmetric states are energetically frustrated and this leads to a competitive energy landscape (see Methods for further details of how this is implemented in energy Hamiltonian) [42], [43], [47]. Our simulation of the Ncd dimer in the absence of the MT reveals both symmetric and asymmetric conformations. Comparison of the contact maps explicitly demonstrates that the pattern of head-stalk contacts in the asymmetric state differs from that in the symmetric state (Figs. 2c and 2d). Interestingly, while each MH can acquire distorted configuration with equal probability, we do not observe a conformation where both MHs are distorted. We found that only one of MHs is distorted towards its asymmetric conformation; the other MH still retains a conformation similar to the one in symmetric dimer. To quantify our findings from simulation, we follow the dynamics of the inter-residue distance (E567 from each monomer) in the dimer structure (Fig. 3a). The distribution of inter-residue distance shows two peaks corresponding to the symmetric and asymmetric conformations with preference to the symmetric conformer. Since the probability for a monomer to sample one of the two asymmetric states is small (prob. = exp(−2.1)∼0.12 from Fig. 3b), the odds to observe a conformation with both MHs being distorted are even smaller (Fig. 3b). Two-dimensional free energy diagram (Fig. 3b), plotted using the distances between K325 and E567 of the two monomers, shows that there are three free energy minima; one for the symmetric state, the others for the two asymmetric states (asymmetric A and B in Fig. 3b). It is also evident from the free energy diagram that a configuration with both heads being distorted is inaccessible.

In addition, principle component analysis [48] of our simulation trajectories (see Methods) reveals that the first two low frequency modes are associated with symmetry-breaking fluctuations that are responsible for bringing the symmetric Ncd structure towards the asymmetric state (Fig. 3c) (also see the supporting movie S1).

### Distorted MH has nonnative-like environment in the NT binding pocket

**Figure 4:**
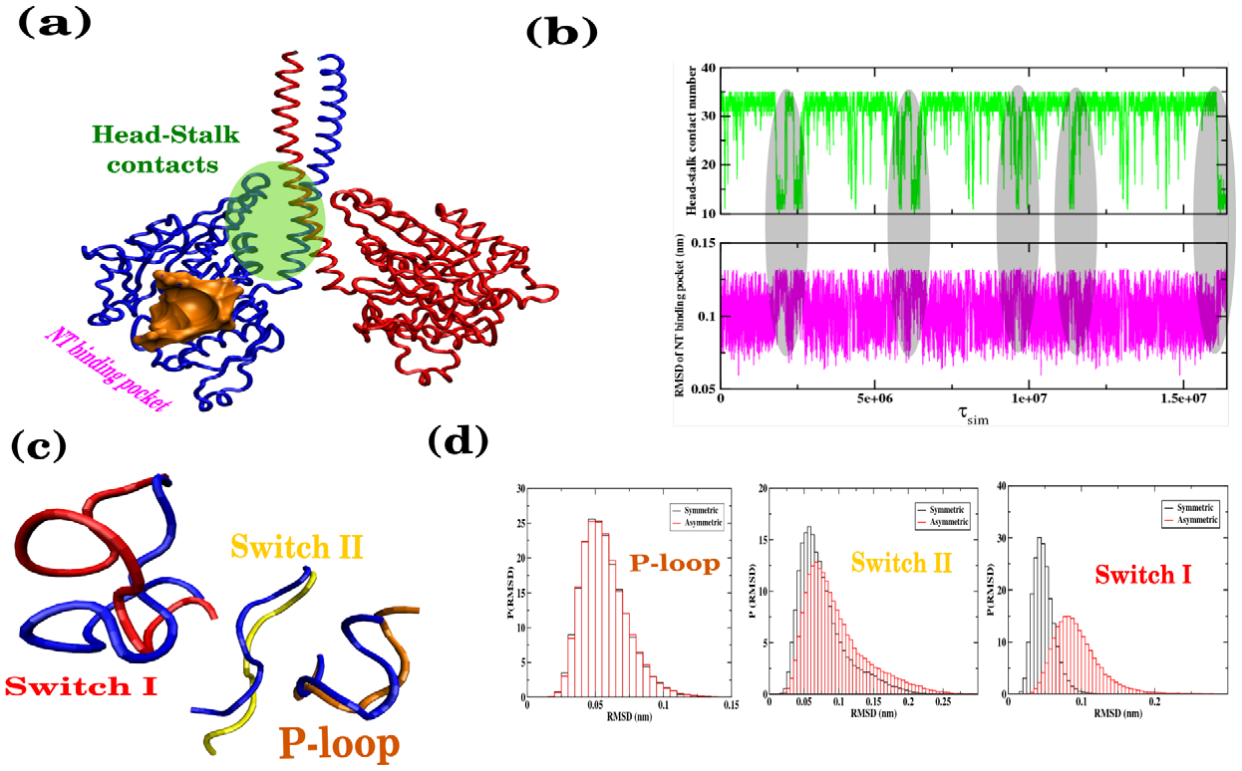
Correlation between symmetric-asymmetric transition and fluctuation at NT binding region. (a) Head-stalk contact region and NT binding pocket in Ncd motor are marked on the Ncd structure. (b) Disruption of the head-stalk contact induces disruption of NT binding pocket. Shaded in grey are the anti-correlated signals between the number of head-stalk contacts and the RMSD of NT binding pocket. (c) Different elements of NT binding pocket region in Ncd motor are marked: P-loop (orange), switch I (red) and switch II (yellow). Structural differences of these motifs are compared between the two states. For symmetric state, we use blue color for all elements. Switch I region reveals maximum deformation. (d) RMSD distribution of P-loop, switch I and switch II are shown for both the states. Note the noticeable difference for switch I region.

To identify the correlation between the MH distortion and the NT binding affinity, we first monitor the change in the number of the head-stalk contacts and the RMS deviation of the NT binding pocket (residues 432–451, 535–555, 576–590) (Fig. 4a). Our simulation finds an interesting anti-correlation between these two quantities at the global level when the distortion dynamic between the symmetric and asymmetric conformers take place (Fig. 4b). As the head-stalk contacts in the symmetric state decrease, the RMS deviation of the NT binding pocket region increases. This anti-correlation is observed in both heads, suggesting that there is an allosteric communication between the head-stalk contact region and the nativeness of the binding pocket. Furthermore, inspecting the details of NT binding pocket, i.e. (residue 434–441 P-loop (residue 434–441), switch I (residue 540–549) and switch II (residue 580–585) [49] in both symmetric and asymmetric states, we find that there is a noticeable enhancement of fluctuation in switch I region whereas the change in the fluctuation dynamics of switch II and P-loop is relatively small (Fig. 4c, 4d). This provides is a clear signature of the increased distortion (especially for switch I) of the ADP binding pocket when it acquires an asymmetric conformation. It may be argued that there is an allosteric communication between the head-stalk contact region and ADP binding pocket as symmetric-asymmetric transition involves changes in head-stalk contact pattern. Provided that the distortion in the NT-binding pocket leads to a weakening the ADP binding affinity [18], [19], our simulation results suggest a possible explanation for the origin of the biphasic ADP release kinetics observed in Ncd dimer in solution and also confirm the proposition by Foster *et al* [28] about an apparent asymmetry between the two MHs of the Ncd dimer.

### Population shift mechanism of microtubule binding

Based on our above findings, we also propose that the population shift (or conformational selection) mechanism is involved when Ncd binds to MTs. In the solution, Ncd can have a stable symmetric conformation with an asymmetric conformation. However, because the NT free-ADP (φ-ADP) state, attained due to fast ADP release from asymmetric conformation [26], is a strong MT-binder, the equilibrium population associated with asymmetric ADP-ADP state in solution would be depleted as more Ncd motors bind to MTs. This MT-binding process produces a flux of population shift from the stable symmetric conformation to the less stable asymmetric conformation.

### Coiled-coil stalk in φ-ADP state is plus-end-directed

**Figure 5:**
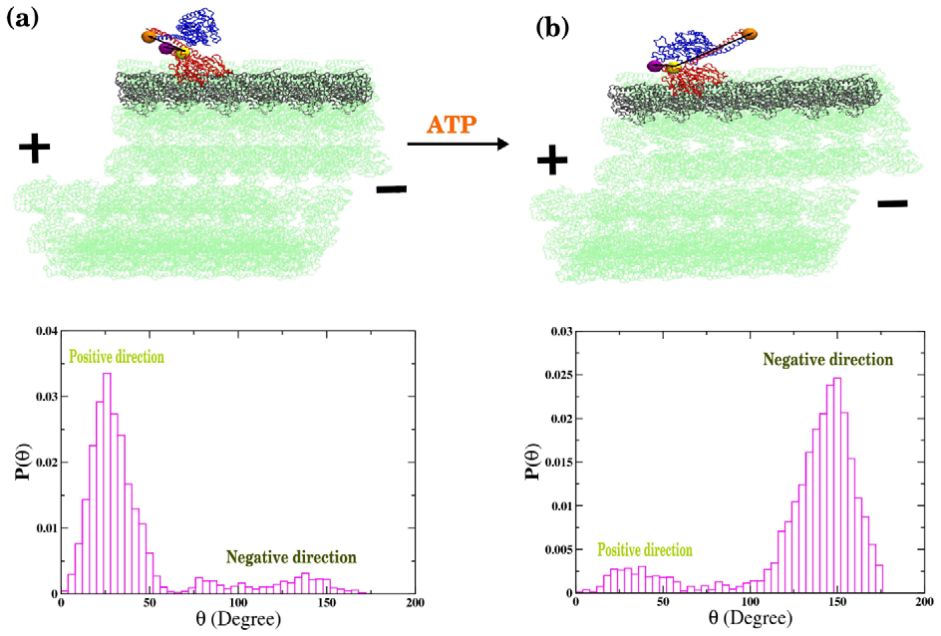
ATP dependent population change and minus-end directed power stroke of Ncd on MT track. (a) Distributions of the angle (θ), among LEU296 in the unbound monomer and D424, E567 in the bound monomer, calculated from simulation for φ-ADP state on MT. The structure of Ncd with *θ*∼25° (peak position) is depicted on the MTs. (b) The angle (θ) distribution calculated from simulation for the ATP-ADP state. The structure of the Ncd on the MT with *θ*∼150° (peak position) is shown on the top. Change from φ-ADP to ATP-ADP state (a→b) implies the minus-end-directed power stroke.

Next, we perform the simulation of φ-ADP state of Ncd dimer with the NT free head being bound to the MT. Since no crystal structure is available in the protein data bank (PDB) for the MT bound Ncd, we use the binding interface (contacts) between MT and kinesin-1 by noting that MH of Ncd is homologous to that of kinesin-1. This is also supported by docking studies that revealed similar orientation of the binding head for these two motors [25]. Thus, by incorporating the MH-MT contacts in our structure-based model we simulated the φ-ADP state on the MTs. Our simulation finds that the Ncd on the MT (Fig. 5a) prefers the symmetric conformation whose coiled-coil stalk points towards the plus-end, which is in agreement with the previous Cryo-EM measurement [27]. The distribution of an angle *θ*, defined using three residues (LEU296 in the unbound monomer and D424, E567 in the bound monomer; see Fig. 5) is bimodal with a dominant population near *θ*∼25° for symmetric and a small population near *θ*∼150° for asymmetric states, respectively. Of particular note is that the interaction of Ncd with MTs greatly reduces the population of asymmetric state in comparison to that of the solution state (compare Figs. 3a and 5a).

### Coiled-coil stalk in ATP-ADP state directed towards minus end of microtubule

Subsequently, we perform the simulation of the MT bound Ncd dimer in the ATP-ADP state. Several mutation studies have suggested the importance of the neck-junction region for the directional motility of Ncd dimer [32], [33], [50]. Bioinformatics analysis based on COILS [50], which assess the feasibility of the amino-acid sequence to form a coiled-coil structure, have also reported that the contacts near the neck-junction region are metastable [50], [51]. Additionally, binding of ATP to the NT free MH may allosterically influence the residues in the junction region. Thus, to mimic the effect of ATP binding we increase structural flexibility by destabilizing the contacts in the junction region (see Methods for details). Our simulation renders a striking inversion from a symmetric to an asymmetric biased landscape (Fig. 5). The distribution of the angle (θ) calculated above for the φ-ADP state (Fig. 5b) now shows a peak around 150°, suggesting that the Ncd dimer stays mostly in the asymmetric conformation when the neck-junction becomes flexible. This finding is in agreement with the previous cryo-EM study that the ATP-ADP state has a coiled-coil stalk pointing towards minus-end of MTs [27]. Thus, our simulation indicates a direct link between destabilization of head-stalk contacts and stabilization of the asymmetric Ncd on MTs. In fact, the conformational change of Ncd from symmetric to asymmetric state leads to the swinging of coiled-coil stalk from plus-end to minus-end, accounting for the structural origin of minus-end directionality of Ncd motors on MTs.

## Discussion

**Figure 6:**
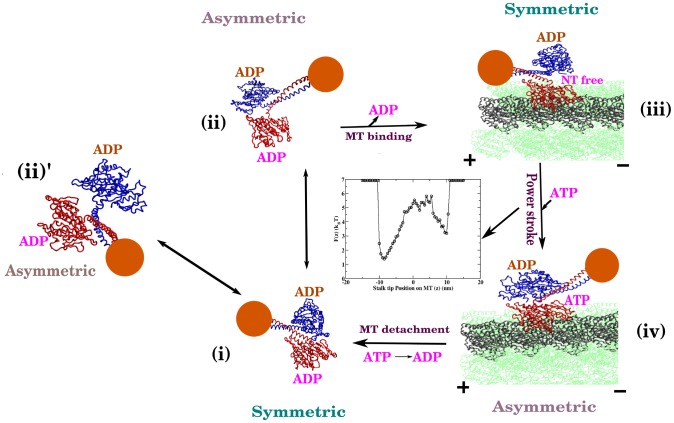
Mechanochemical cycle for Ncd motor. In solution, Ncd exists in (i) symmetric and two asymmetric states (either (ii) or (ii)’). On the MTs, Ncd in φ-ADP state exists predominantly in (iii) symmetric plus-end directed conformer. ATP binding stabilizes (iv) asymmetric conformer, which gives rise to minus-end-directed stroke. Finally, the ATP hydrolysis returns the Ncd to the ADP-ADP state that has weak MT-binding affinity; thus Ncd dissociates from MT to the solution. The free energy (F(z)) profile of the power stroke step which is calculated by taking stalk tip position (z) on MT as order parameter is also shown in the same figure.

The results obtained from our simulation can be recapitulated in the mechanochemical cycle for Ncd motor in Fig. 6. In solution, the Ncd dimer (ADP-ADP state) exists in either (i) symmetric or (ii) (or (ii)’) asymmetric conformations with more dominant population in the symmetric state. In the asymmetric conformation, distortion in one of the heads from its wild type structure is correlated with enhanced fluctuation near ADP binding pocket. Hence, the distorted head in an asymmetric conformation has a lower ADP affinity that facilitates the ADP release. Simulations from our Ncd model explicitly demonstrate that this symmetric-to-asymmetric conformational transition occurs occasionally and that there is a clear correlation between this transition and the increase of fluctuation at the binding pocket of the distorted head (especially in switch I) [49]. This transition explains how the bi-phasic ADP release kinetics occurs while Ncd is a homodimer. Following, the NT-free MH, which retains strong MT-binding affinity (K_d_∼nM), binds to the MTs and reaches (iii) a further stabilized symmetric conformation on the MT, whose coiled-coil stalk points towards the plus-end. (iv) ATP binding to the free head destabilizes the neck-head junction region of the MT-bound head, which overturns the ensemble of symmetric Ncd conformation in the φ-ADP or ADP-ADP state into the ensemble of asymmetric state that has a minus-end-directed coiled-coil stalk. Our studies have identified head-neck junction region as the critical structural element responsible for power stroke mechanism of Ncd, which in fact is amenable to further experimental studies. Furthermore, the step involving (iii)→(iv) corresponds to the power stroke, i.e., the force production due to conformational changes. Our estimate of a lower bound of stall force from a probability *P*(*z*), which provides a potential of mean force *F*(*z*) = −*k*_B_ *T* log *P*(*z*) (see the inset between step (iii) and (iv) in Fig. 6), where z is the position of stalk tip projected along the MT axis is 0.42 pN. Compared to the stall force value of other molecular motors (2–3 pN for myosin V (Biophys J. (2000) 79:1524–1529, PNAS (2004) 101:5542–5546) and 5–6 pN for kinesin-1 [7]), this value is rather small; however, given that the Ncd is a non-processive motor that should cooperate with other motors to generate force, our estimate of the stall force for a single Ncd, which no experiment has measured to date, is not unreasonable. Finally, the ADP-ADP (or ADP·P_i_-ADP) state of the Ncd with weak MT affinity (K_d_> mM) [52], produced as a result of ATP hydrolysis and subsequent release of inorganic phosphate from the bound head, leads to the dissociation of the Ncd motor from the MT ((iv)→(i)). The minus-end-directed power stroke of Ncd motor is generated between the step (iii) and (iv). The dissociation of Ncd motor after a single stroke suggests that the movement of Ncd on the MTs is non-processive. Thus the minus-end-directed cargo transport is realized by cooperation between multiple Ncd motors [26].

The results of the simulation can be understood as follows. In the case without MT, both the symmetric and asymmetric states were populated as we have constructed a dual-basin structure-based model with a bias towards the symmetric state. The symmetric state is more populated because the native bond, angle and dihedral parameters are derived from symmetric crystal structure (see Methods). In the MT bound φ-ADP state, the presence of MT reduces the configuration space of the Ncd. This increases the stability of symmetric state whose coiled-coil stalk points towards the plus end of MT. Binding of ATP to the empty head, destabilizes few contacts in the head-stalk junction region which in turn destabilizes the symmetric basin. Therefore, it now preferentially populates the asymmetric basin whose coiled-coil stalk points towards the minus end of MT.

Molecular motors in the kinesin superfamily share the common structural motifs, such as the MH, the neck-linker (or the extended coiled-coil stalk), and the neck-helix that are used in their biological functions. The MH domain used for MT-binding is essentially identical for kinesin-1, Ncd, Eg5 that use tetrameric MHs (two pairs of dimer heads) to bind the two anti-parallel MTs, and other kinesin-like proteins. Furthermore, the MT-binding affinity of MH in Ncd obeys a similar rule as in kinesin-1; the MH in ADP state has weak MT-affinity whereas the MH in φ or ATP state binds MT strongly [23], [53], [54], [55]. Although there is a quantitative difference in the value of the binding affinity for different kinesin families due to the sequence variation, kinesin-1 and Ncd have the identical “footstep” on MTs and bind to MTs tightly during the processes of ATP binding and hydrolysis. Our study asserts that the determining factors of the directionality in kinesin-1 and Ncd are the structural motifs peripheral to the MH (Fig. 1d), i.e., neck-linker and neck-helix. This is evident from our Ncd model that is adapted from the previous model for kinesin-1. The difference in directionality for the kinesin-1 and the Ncd is a remarkable example of how motor proteins in the kinesin superfamily diversify their functions by repositioning the structural motifs peripheral to the MH domain that are in charge of catalytic activity [31]. Our study provides unambiguous structural and dynamical understandings to the effect of such repositioning on the biological function.

## Methods

Simulations were performed using standard structure-based (SB) potential in SMOG [56] protocol implemented in Gromacs. MT bound structure was generated by overlapping the MH of Ncd on the single-headed kinesin (KIF1A) bound structure on tubulin (PDB ID 1IAO). We used 13-protofilament structure [57] to illustrate MT bound Ncd structure.

### Structure-based potential

Using the MT bound Ncd structures generated by aligning the crystal structures of Ncd on MTs, we simulated Ncd motor dimer with or without the MT track. Langevin dynamics simulations using the SB potential were performed where equation of motion was integrated using a Verlet algorithm. The energy function used for these simulations are given as follows:
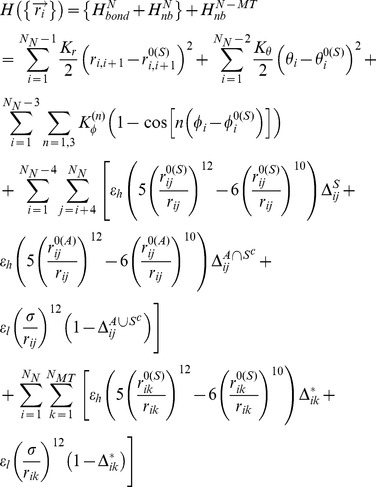



Here, total energy Hamiltonian is separated into intramolecular interaction for the Ncd and intermolecular interaction at the Ncd-MT interface. The subscripts N and MT denote the Ncd and MT, respectively and N-MT denotes the Ncd-MT interaction. We have fixed coordinates of MT in space throughout the simulation.

The first and second terms associated with bond potential in 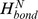 define the backbone interactions. The bond distance (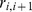) between the neighboring residues *i* and *i*+1 are constrained harmonically with respect to its native bond distance (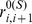) with a spring constant (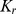) of 20 kcal/(mol×Å^2^). In the second term, the angle 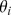 defined among residues *i*, *i*+1 and *i*+2 is constrained harmonically around its native state value (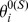) with 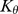 = 20 kcal/(mol×rad^2^). The third term represents the dihedral angle potential with 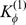 = 1.0 kcal/mol and 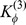 = 0.5 kcal/mol that describes the rotation of the backbone involving successive residues from *i* to *i*+3. The native values, 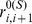, 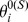, and 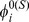 are taken from the symmetric conformer and the superscript (*S*) refer to the symmetric state.

The 10–12 Lennard-Jones (LJ) potential is used in 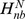 to describe the interactions that stabilize the non-bonded native contacts. A native contact is defined for a pair of residues (*i* and *j*), if the distance between them is less than 8 Å in the native state and 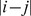
>3. If *i* and *j* (*k* for Ncd-MT interface) residues are in contact in the native state, 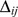 (or 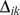) = 1; otherwise 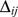 (or 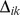) = 0. It is of particular note that this non-bonded LJ potential retains three terms [42], [43]: (i) the native bias mainly from symmetric state (the first term) with an equilibrium distance 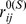 for *i* and *j* residue pairs, (ii) the perturbative bias from asymmetric state (the second term) with an equilibrium distance 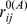, and (iii) the repulsive potential for the residue pairs that belong to neither the symmetric nor the asymmetric state. The superscript of 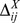, i.e., *X* = 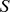, 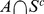, 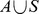 and, refers to the symmetric state, “purely” asymmetric state, and the union of symmetric and asymmetric state, respectively, so that 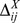 = 1 when *i* and *j* residue pairs are in the state *X*, otherwise 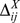 = 0. Non-native pairs with 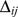 (or 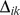) = 0 are under repulsive potential with a distance parameter σ = 4 Å. We assign ε_h_ = 1.8 kcal/mol for the upper part of intraneck, interneck helix (residue <331) and Ncd-MT interactions to secure coiled-coil association and MT binding, respectively. For other residue-residue interactions, we set ε_h_ = ε_l_ = 1.0 kcal/mol regardless of sequence identity. The parameters determining the native topology 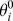, 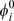, 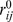 and 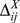 are determined from the symmetric and asymmetric crystal structure of Ncd.

The intramolecular non-bonding energy term, 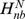, provides competition between the native contacts from symmetric and asymmetric states with more bias towards the symmetric conformer when NT-binding pocket is in φ or ADP state. This bias changes to the asymmetric state by removing a few native contacts from the neck-stalk junction (see below).

### Simulations

Initial structures in each case were relaxed under the SB Hamiltonian and subsequently Langevin dynamics simulations at low-friction limit were performed at T = 300 K to sample the equilibrium structural ensemble. The equation of motion for the Langevin dynamics used for integration is
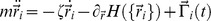
where ζ is the friction coefficient, 
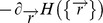
 is the conformational force. 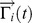 is the random force satisfying 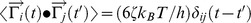 where integration time *h* is discretized. In this dynamics we chose ζ = 0.05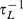 and *h* = 0.0025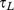 with 
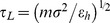
. Low friction was chosen for the purpose of effective conformational space sampling [58].

### ADP-ADP state

To simulate the Ncd dimer in solution we used the topological information from the crystal structures with PDB code of 1CZ7 (symmetric state) and 1N6M (asymmetric state). All the native backbone and dihedral parameter was derived from the symmetric crystal structure. For each monomer nonbonded potential corresponding to the additional contacts for the asymmetric conformation are added to the potential. Here, we neglected the Ncd-MT interaction term from the Hamiltonian to simulate Ncd dimer in solution without MT.

### Principle component analysis

We superimposed the structure of each frame from the simulation onto the symmetric dimer to obtain the covariance matrix [48]. After diagonalization we examined the first few low frequency modes. The lowest frequency eigenvector was then represented residue-wise to illustrate the collective dynamics towards the asymmetric state.

### Construction of MT bound state

For the construction of the MT bound state of Ncd, we used three structures: single-headed kinesin (KIF1A) bound to the MT (PDB ID 1IA0); Ncd dimer structure (PDB ID 1CZ7); and three consecutive αβ tubulin dimers from 13-protofilament MT structure [57]. We overlapped chain A of 1CZ7 onto chain K of 1IA0 and αβ tubulin dimer of 1IA0 onto terminal αβ tubulin dimer of the tubular MT structure. This leads to the MT bound state of Ncd. Here, we assume that Ncd employs a similar binding interface on the MTs with kinesin-1, which was shown in earlier studies [26], [55], [59], [60], [61].

### Simulation of φ-ADP state on MT

Using the structure obtained from the construction of the MT bound state of Ncd, we extracted the Ncd-MT contacts at the interface and included in the total Hamiltonian to generate the conformations corresponding to the NT free-ADP state on MT.

### Simulation of ATP-ADP state on MT

It was proposed earlier that the head-stalk junction of Ncd plays important role in determining the directional motility of motor [32], [33], [50]. While retaining the same Hamiltonian as the φ-ADP state on MT, we deleted a few native contacts in the junction region (residues 341–345 of the coiled-coil stalk and residues 346–351 of the MH) between the coiled-coil stalk and bound head of Ncd to mimic the binding of ATP. The junction region (lower part of extended coiled-coil region) of the Ncd motor is shown explicitly in the Fig. 1b.

## Supporting Information

Video S1 The motion along the one of the two low frequency principal component of Ncd is shown in this video.
(MPG)
